# Regional variations in lumbar spine surgery in Finland

**DOI:** 10.1007/s00402-021-04313-0

**Published:** 2021-12-31

**Authors:** Heikki Mäntymäki, Ville T. Ponkilainen, Tuomas T. Huttunen, Ville M. Mattila

**Affiliations:** 1 Department of Orthopaedics and Traumatology, Tampere University Hospital, Teiskontie 35, PL2000, 33521 Tampere, Finland; 2 Department of Anesthesiology, Tampere University Hospital, Teiskontie 35, PL2000, 33521 Tampere, Finland; 3 Faculty of Medicine and Health Technology, Tampere University, Tampere, Finland; 4 COXA Hospital for Joint Replacement, Biokatu 6, 33520 Tampere, Finland

**Keywords:** Lumbar spine surgery, Variation, Surgery rates, Spine register

## Abstract

**Introduction:**

The regional variation in spine surgery rates has been shown to be large both within and between countries. This variation has been reported to be less in studies from countries with spine registers. The aim of this study was to describe the regional variation in lumbar spine surgery in Finland.

**Materials and methods:**

This is a retrospective register study. Data from the Finnish National Hospital Discharge Register (NHDR) were used to calculate and compare the rates of lumbar disc herniation (LDH), decompression, and fusion surgeries in five University Hospital catchment areas, covering the whole Finnish population, from January 1, 1997, through December 31, 2018.

**Results:**

A total of 138,119 lumbar spine operations (including LDH, decompression, and fusion surgery) were performed in Finland between 1997 and 2018. The regional differences in the rate of LDH surgery were over fourfold (18 vs. 85 per 100,000 person years), lumbar decompression surgery over threefold (41 vs. 129 per 100,000 person years), and lumbar fusion surgery over twofold (14 vs. 34 per 100,000 person years) in 2018. The mean age of the patients increased in all regions during the study period.

**Conclusions:**

In Finland, the regional variations in spine surgeries were vast. In a country with a publicly funded healthcare system, this finding was surprising. The recently created national spine register may serve to shed more light on the reasons for this regional variation.

## Introduction

Medical practice variation across regions, hospitals, and physicians is a recognized phenomenon that has been prevalent for a long time [1]. Moreover, trends in medical practice variation have also been shown to be prevalent in surgical treatment [2].

According to several published surgical studies, a large variation in spine surgery rates has been shown to exist both within and between countries [3–5]. In some cases, the reported regional variations have been vast, especially in the US, where spine surgery rates have differed by as much as 20-fold between hospitals [6]. In an international comparison carried out in 1987 and 1988, the spine surgery rates in the US were almost twofold compared to that in Finland, threefold compared to Sweden, and nearly sixfold compared to England. Surgery rates can also vary within a country. For example, in a recent study of surgery rates in Norway, there was a 40% difference between those regions with the highest and lowest rates of lumbar spinal stenosis surgery [7]. In Sweden, the regional differences are also minor [8–10]. The small differences in Norway and Sweden may be explained by both countries having a longer history of national spine registers and the reporting of regional variations [11–13].

A growing body of evidence has served to clarify the indications for surgical interventions of the spine and may have diminished the regional differences in spine surgery rates [14–17]. The Finnish national spine register was created in 2016, and today, it is in use in the largest hospitals in Finland [18]. To date, however, no annual report has been published. The aim of our study was, therefore, to assess the regional variation in spine surgery in Finland between 1997 and 2018 and to benchmark any regional variation prior to the active reporting of the Finnish spine register data.

## Materials and methods

This retrospective register study was based on data collected from the Finnish National Hospital Discharge Register (NHDR) from January 1, 1997, through December 31, 2018. All patient characteristics, such as age, sex, domicile, hospital, primary and secondary diagnosis, surgeons’ specialty and operations performed during the hospital stay, were obtained from the register. In Finland, it is mandatory for all hospitals to collect data for the NHDR, and thus the coverage and accuracy of the database has been shown to be excellent [19–21].

Hospitalizations due to spine surgery were selected using NOMESCO (Nordic Medico-Statistical Committee) classification surgical procedure codes combined with diagnosis codes from the International Classification of Diseases, Tenth Revision (ICD-10) [22, 23]. The codes describing discectomy, decompression and fusion procedures, are: ABC07, ABC16, ABC17, ABC26, ABC36, ABC56, ABC66, ABC99, NAG60-67, and NAG99. The patients who underwent these procedures were classified into four groups according to specific diagnosis codes as described by Salmenkivi et al. [24]: Herniated intervertebral disc (M51.1, G55.1), spinal stenosis, (M47.2, M47.9, M48.0), degenerative disc disease (M47.82, M51.3) and spondylolysis and spondylolisthesis, (M43.0, M43.1).

In case of multiple procedures or multiple coding, such as if a patient underwent fusion and decompression, the operation was considered as fusion. Then again, if a patient underwent multiple similar operations during the same admission, they were considered a single operation. Finally, if there were multiple operations for a single patient within multiple admissions, they were considered as separate operations.

Patients under 18 years of age were excluded. The Finnish public healthcare system is built around five University Hospitals that together form five separate hospital catchment areas: Helsinki, Tampere, Turku, Kuopio, and Oulu. All operations performed in private hospitals were added to analyses and the region was determined based on the municipality of the patient.

### Statistical analysis

The annual operation rates (per 100,000 person years) were calculated based on the entire adult population of each University Hospital catchment area. The data contained annual midyear populations and it were obtained from the national population register (the Official Statistics of Finland). All analyses were performed using R version 4.0.2 (R Foundation for Statistical Computing, Vienna, Austria).

## Results

Data was collected of 138,119 patients with lumbar spine operations (including LDH, decompression, and fusion surgery). Altogether, 17,091 (12%) operations were performed in private hospitals, whereas 121,028 (88%) of the operations were performed in public hospitals. After targeting the surgical procedure codes to specific diagnosis codes, as described in “Materials and methods”, 133,913 operations were included to analyses.

**Fig. 1:**
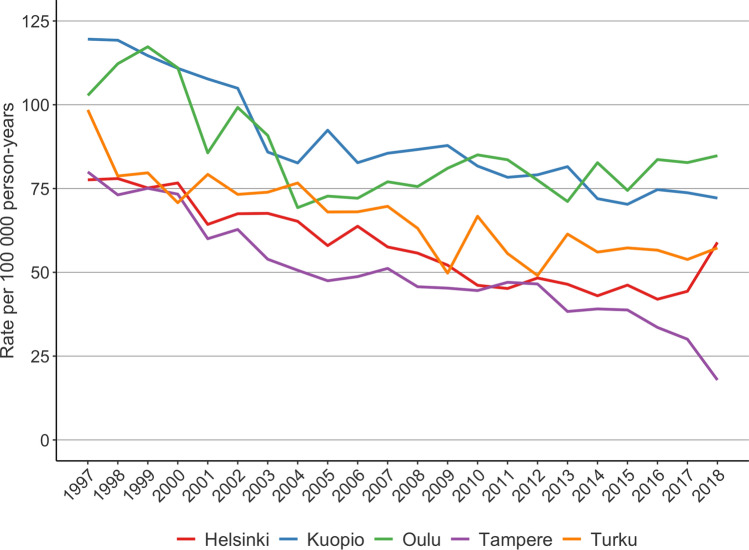
Regional rates of lumbar disc herniation surgery in Finland from 1997 to 2018

The number of LDH operations was 60,918 between 1997 and 2018. The annual rate of LDH surgery decreased in all five University Hospital catchment areas during the study period. The rate was higher in the northern catchment areas of Kuopio and Oulu compared to the other three University Hospital catchment areas (Fig. 1). The annual operation rate (per 100,000 person years) decreased by 24% (from 78 to 59) in the Helsinki University Hospital catchment area, by 78% (from 80 to 18) in the Tampere University Hospital catchment area, by 42% (from 98 to 57) in the Turku University Hospital catchment area, by 40% (from 120 to 72) in the Kuopio University Hospital catchment area, and by 17% (from 103 to 85) in the Oulu University Hospital catchment area.

**Fig. 2:**
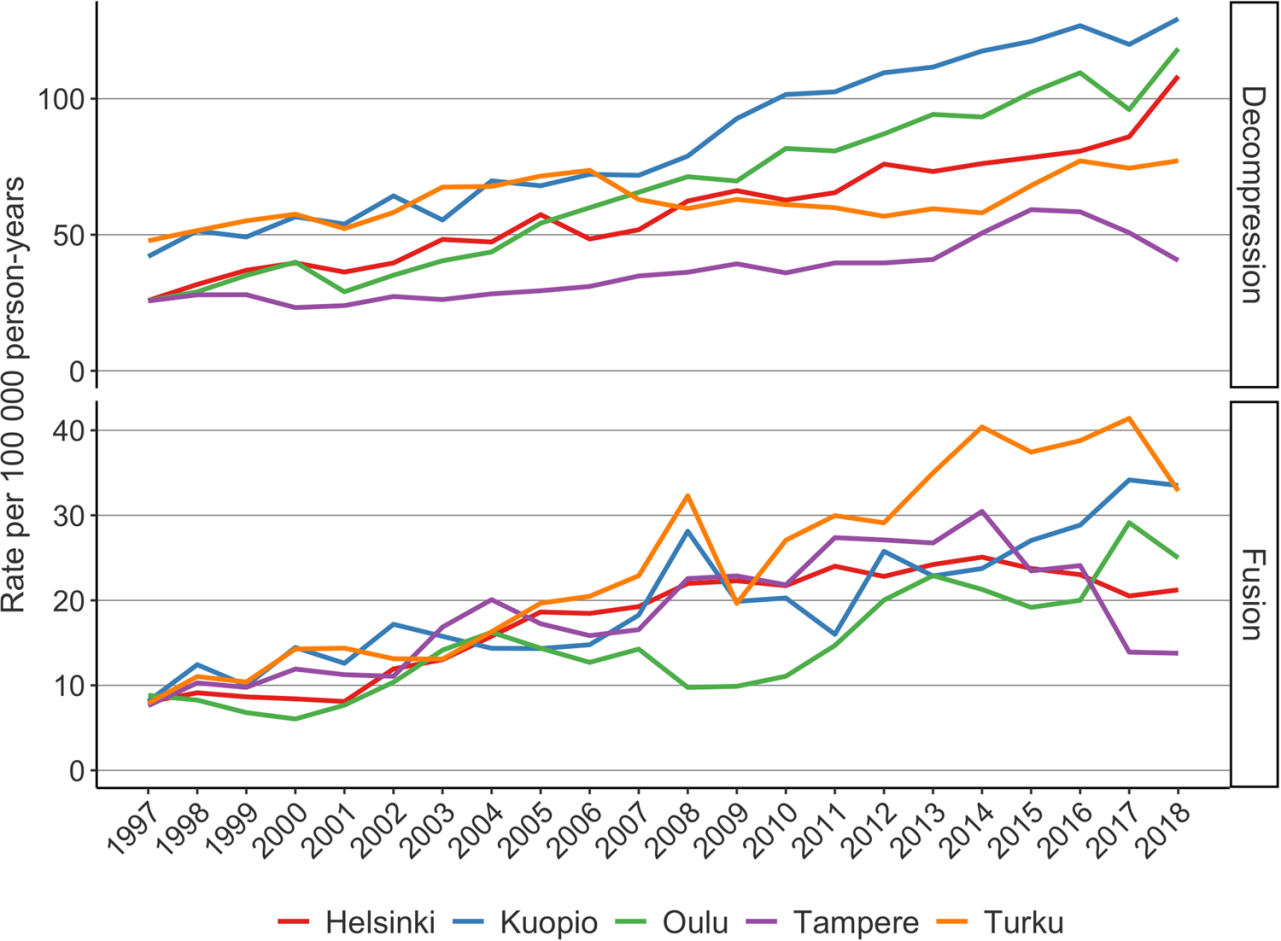
Regional rates of lumbar decompression and lumbar fusion surgery in Finland between 1997 and 2018

The rates of decompression surgery increased in all five University Hospital catchment areas between 1997 and 2016 (Fig. 2). The operation rates continued to increase after 2016 in all regions, with the exception of the catchment area of Tampere University Hospital and Turku University Hospital. Between 1997 and 2018, the annual operation rate (per 100,000 person years) for decompression surgery increased by 315% (from 26 to 108) in the Helsinki catchment area, by 58% (from 26 to 41) in the Tampere catchment area, by 60% (from 48 to 77) in the Turku catchment area, by 207% (from 42 to 129) in the Kuopio catchment area, and by 354% (from 26 to 118) in the Oulu catchment area.

**Fig. 3:**
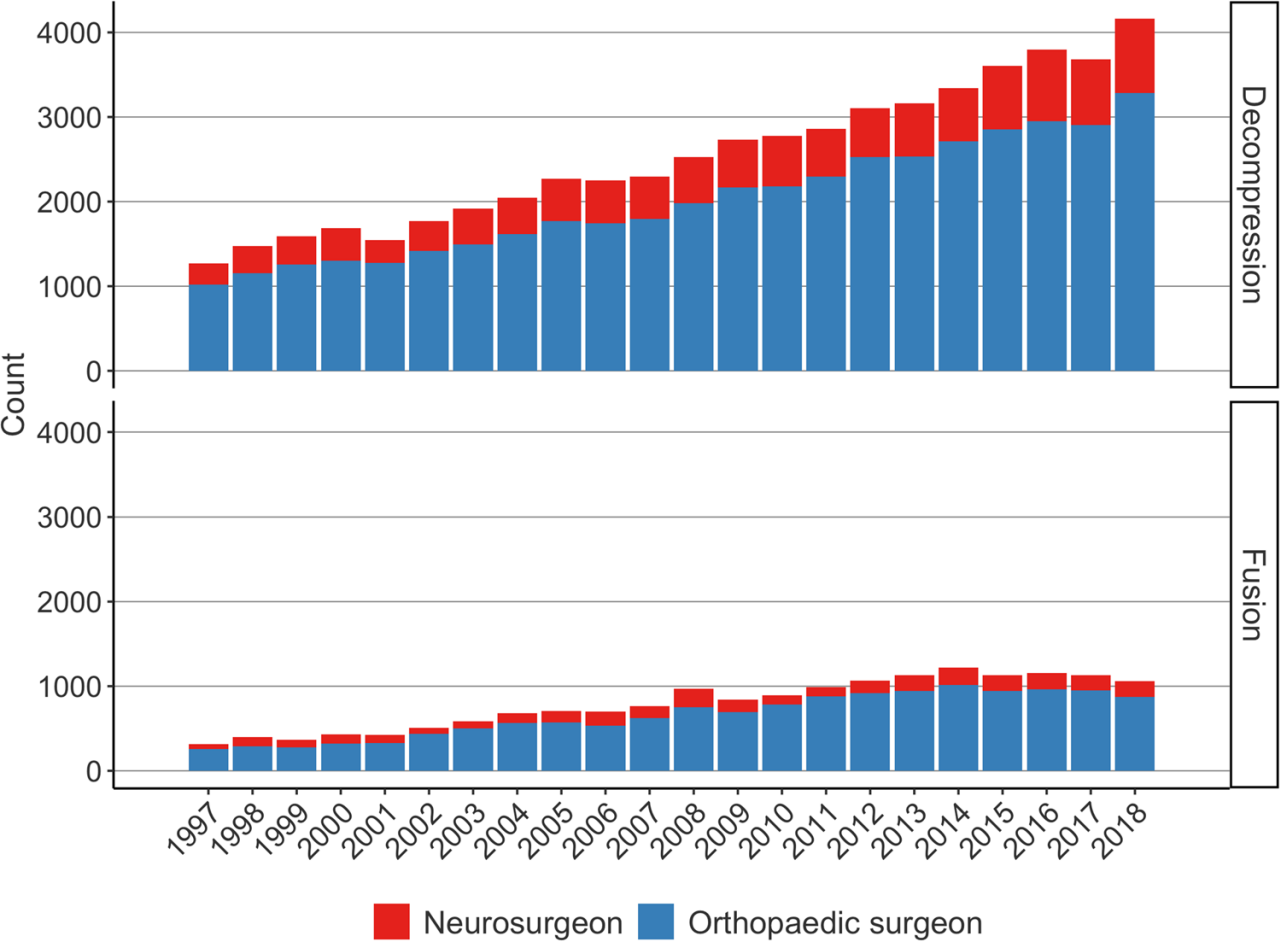
Number of lumbar decompression and lumbar fusion operations performed in Finland between 1997 and 2018 with proportional distribution between neurosurgeons and orthopaedic surgeons

The rate of lumbar spine fusion increased in all five University Hospital catchment areas between 1997 and 2014. After 2014, however, the rate in Helsinki, Tampere, and Turku decreased (Fig. 2). Between 1997 and 2018, the annual operation rate (per 100,000 person years) for fusion surgery increased by 162% (from 8 to 21) in the Helsinki catchment area, by 75% from (8 to 14) in the Tampere catchment area, by 313% (from 8 to 33) in the Turku catchment area, by 325% (from 8 to 34) in the Kuopio catchment area, and by 178% (from 9 to 25) in the Oulu catchment area. The proportions of decompression and fusion surgeries performed by orthopaedic surgeons and neurosurgeons remained stable (Fig. 3).

**Fig. 4:**
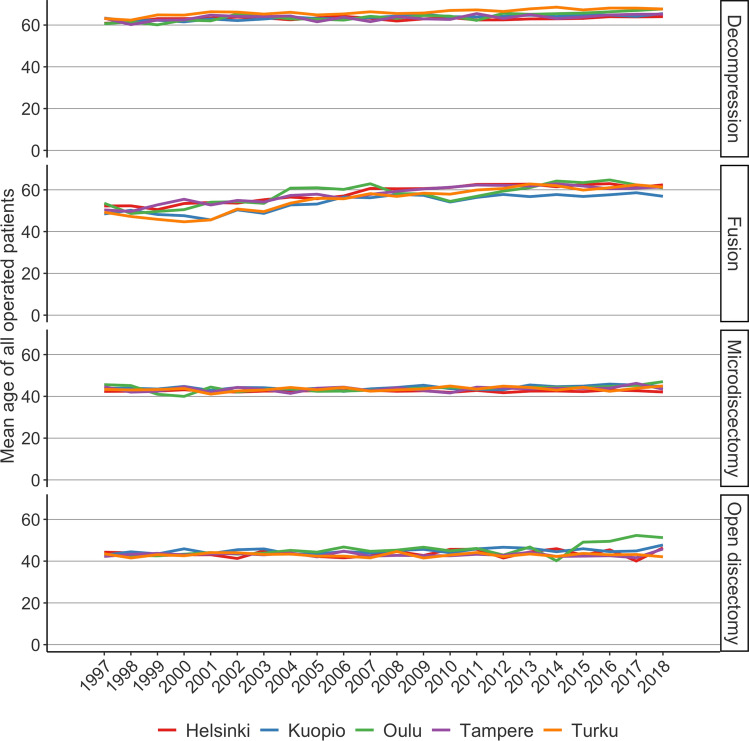
Mean age of patients undergoing lumbar spine surgery in Finland between 1997 and 2018

The mean age of patients undergoing lumbar spine surgery between 1997 and 2018 increased steadily in all five University Hospital catchment areas (Fig. 4). The increase in mean age was 10 years (from 47 to 57) in the Helsinki catchment area, 11 years (from 48 to 59) in the Tampere catchment area, 8 years (from 50 to 58) in the Turku catchment area, 9 years (from 49 to 58) in the Kuopio catchment area, and 12 years (from 47 to 59) in the Oulu catchment area.

The change in mean age of patients undergoing LDH surgery from 1997 to 2018 was minor. In Helsinki, the mean age was 43 in both 1997 and 2018. The mean age increased from 43 to 44 years in Tampere, from 43 to 44 years in Turku, from 43 to 46 years in Kuopio, and from 43 to 47 years in Oulu*.* The increase in mean age was more distinct in decompression and fusion surgery. In decompression surgery, the mean age increased in Helsinki from 61 to 64 years, in Tampere from 63 to 65 years, in Turku from 63 to 68 years, in Kuopio from 63 to 65 years, and in Oulu from 61 to 68 years. In fusion surgery, the mean age increased in Helsinki from 52 to 62 years, in Tampere from 50 to 61 years, in Turku from 49 to 61 years, in Kuopio from 48 to 57 years, and in Oulu from 54 to 61 years.

## Discussion

The main finding of the present study was the notable differences in surgical activity between the five Finnish University Hospital catchment areas. In 2018, the regional differences in the rate of LDH surgery was over fourfold, lumbar decompression surgery over threefold and lumbar fusion surgery over twofold. The mean age of the patients increased in all regions during the 1997 to 2018 study period. The proportions of spine surgeries performed by neurosurgeons and orthopaedic surgeons remained stable during the same period. Taking into account that the healthcare system in Finland is publicly funded, the finding that notable differences exist between different hospital catchment areas was surprising.

Seitsalo et al. [25] reported a variation in the rates of spine surgery in Finland from 1987 to 1994. The regional variation in LDH surgery was between 34 and 143 (per 100,000 person years) procedures. In other words, there was a more than fourfold regional variation between the highest and lowest rates in LDH surgery. Furthermore, a higher rate of LDH surgery was found in the northern and eastern parts of the country in their study. This corresponds to the results of the present study as the difference in LDH surgery is still fourfold.

It is unclear why there are still regional differences in the rates of spine surgery in Finland. There is a national guideline in Finland concerning the diagnostics and treatment of low back pain [26]. Conservative treatment is recommended as the primary treatment in patients with radiating low back pain without alarming symptoms, e.g., cauda equina syndrome, suspicion of spondylodiscitis, malignancy or other severe illness. Radiological evaluation of the spine is first recommended after 6 weeks to 3 months of conservative treatment. It is known that an increase in imaging of the spine has an increased impact on overall surgery rates [27]. In Finland, however, the guideline for spinal imaging is consistent and should, therefore, not affect the regional differences in the rates of spine surgery.

The main reasons for the variation in operation rates have been suggested to be a lack of evidence, surgeons’ opinions, and the local cultural practice [6]. In Finland education for spine surgeons is mostly based on local curriculum and a systematic national education is lacking. The public healthcare system tends to offer resources quite evenly throughout the country. The choice for surgical treatment should be based solely on the best available evidence. However, the allocation of resources can vary between specialties within the hospitals, reflecting the values and treatment cultures of different specialties. In addition, it is possible that there is some regional variation in the number of spine surgeons in Finland. The division of work between neurosurgeons and orthopedic spine surgeons also vary regionally and this may further emphasize different cultures in indications of surgical treatment. Furthermore, Northern–Eastern Finland bear greater morbidity burden in general compared to Southern–Western Finland and it is comprehensively reported [28]. Regional difference in morbidity might affect also the spine surgery rates, but it unlikely explains the whole over fourfold difference in operation rates seen in our study.

Keller et al. studied the relationship between rate variations and the outcomes of surgical treatment for LDH and spinal stenosis. They found that the outcomes of spine surgery were better among lower population-based rates. In addition, improvements were seen in Roland disability score, quality of life, and satisfaction [29]. It is possible that in areas with lower operation rate, patients with more severe symptoms are selected for operative treatment and, therefore, benefit more from treatment. Indeed, in the study by Keller et al. [29], the patients in the higher rate areas had fewer severe symptoms and findings at baseline. However, the goal in surgical decision making is to find those patients who will benefit the most from surgery, and the risk–benefit ratio is always going to be more or less a philosophical question. How much benefit should be assumed with a certain amount of risk? We really do not know if more or less is better in the rates of spine surgery.

In Norway, the mean surgery rates in lumbar spinal stenosis varied from 23 (per 100,000 person years) to 32 (per 100,000 person years) between 1999 and 2013, i.e., the regional variation in operation rates only differed by a factor of 1.4 [7]. In Sweden, there were only minor regional differences in the rates of LDH surgery between 1987 and 1999 [9]. Jansson et al. [8] also reported the rates of spine surgery in patients with lumbar spinal stenosis in Sweden between 1987 and 1999. The regional variation in operation rates was from 6 to 13 (per 100,000 person years). The smaller differences in Norway and Sweden may be explained by a longer history of spine registers and reporting of regional variations [10–13, 30]. When comparing the regional variations in spine surgery in the US and Scandinavia, the differences are notable. Weinstein showed high regional differences in the US between 1992 and 2003 as lumbar discectomy and laminectomy rates varied from 60 to 480 (per 100,000 person years) and fusion operations varied by more than 20-times between different regions ranging from 20 to 460 (per 100,000 person years) operations [6]. However, it must be noted that the healthcare system in the US is not publicly funded and this may affect variation between the operation rates.

The increase in the mean age of patients undergoing spine surgery reported in the present study has also been reported in previous studies. Martin et al. studied the trends in fusion procedures in the US between 2004 and 2015 and found a clear shift towards an older population. The mean age of patients undergoing elective lumbar fusion increased from 55 to 60 years [31]. Grovle et al. [7] reported an increase in mean age from 62.6 to 67.2 years in patients undergoing surgery for lumbar spinal stenosis surgery in Norway between 1999 and 2013. Jansson et al. [8] reported the results of spinal stenosis surgery in Sweden. They reported that the mean age increased from 60.1 to 66.8 years between 1987 and 1999. Results from England are similar. Sivasubramaniam et al. [32] concluded that the mean age of patients undergoing surgical procedures for degenerative lumbar spine disease increased from 49.4 in 2000 to 52.3 years in 2013.

In most western countries, there are usually two different departments and specialities, neurosurgeons and orthopaedic surgeons, who perform spine surgery. In Finland, neurosurgeons are more focused on the cervical spine, intradural pathologies, and LDH/decompression surgery, and orthopaedic surgeons focus more on fusion surgery and deformity surgery. However, co-operation between these two departments is also increasingly in Finland. In the study by Seitsalo et al. [25], an increase in LDH surgery was seen between 1987 and 1994, especially among operations performed by neurosurgeons. In our study, the proportional difference between orthopaedic surgeons and neurosurgeons performing lumbar spine surgery remained stable between 1997 and 2018. In Finland, collaboration between the specialities and the findings of the previous study by Seitsalo et al. [25] might have had an impact on flattening the differences between the specialities.

The strength of our study is the comprehensive data from the NHDR that included all spine operations performed in Finnish public hospitals. The coverage and accuracy of the NHDR database is excellent [19–21]. Since 2014, it has been possible for patients to be referred to another hospital catchment area for treatment, if they so request. However, the number of these patients has remained relatively low, less than 1%, and did not, therefore, affect our results.

## Conclusion

The results of our study show regional variation in lumbar spine surgery in Finland. In 2018, the regional differences in the rates of LDH surgery were over fourfold, lumbar decompression surgery over threefold, and lumbar fusion surgery over twofold. Patient age did not differ between catchment areas. The reason for this observed geographical difference may be related to local cultural practice, since resources do not explain this difference in a publicly funded healthcare system. The recent creation of a national spine register may shed more light on these notable differences in surgical activity.
